# Morphological diversification among island populations of intertidal mites (Acari, Oribatida, Fortuyniidae) from the Galápagos archipelago

**DOI:** 10.1007/s10493-017-0149-3

**Published:** 2017-06-20

**Authors:** Tobias Pfingstl, Julia Baumann

**Affiliations:** Institute of Zoology, University of Graz, Universitaetsplatz 2, 8010 Graz, Austria

**Keywords:** Speciation, Multivariate analyses, Dispersal, *Alismobates*, *Litoribates*

## Abstract

The intertidal oribatid mite species *Alismobates galapagoensis* and *Litoribates caelestis* occur on the archipelago of Galápagos. To test for morphological variation between populations of different islands, a comprehensive morphometric study was performed. Four *A. galapagoensis* populations from the islands Bartolomé, Isabela, Santa Cruz and San Cristobal, as well as two *L. caelestis* populations from Bartolomé and Santa Cruz were investigated. The *L. caelestis* populations did not show any significant differences whereas the *A. galapagoensis* populations exhibited clear divergences indicating speciation. Differences in overall size of *A. galapagoensis* apparently followed a gradient from East to West, with specimens from San Cristóbal being the largest and individuals from Bartolomé and Isabela being the smallest. Apart from size, significant shape differences were found in the epimeral region and females showed stronger variation among islands than males. The degree of morphological divergence seems to correlate with geographic distance, i.e. populations from islands located closer to each other showed fewer differences than populations from distant islands. Based on this correlation we suggest that transport between islands has happened mainly by drifting on ocean currents.

## Introduction

The family of Fortuyniidae represents a group of oribatid mites dwelling exclusively in littoral habitats, as for example rocky shores, boulder beaches or mangrove forests. They show a transoceanic distribution but are restricted to shorelines of warm subtropical and tropical areas (e.g. Schuster 1989; Pfingstl and Schuster 2014).

The Eastern Pacific archipelago of Galápagos lies at the equator and offers ideal climatic conditions for these warm-adapted intertidal mites. For that reason, it was not surprising when Schatz (1998) demonstrated that two fortuyniid species have successfully colonized the coasts of this famous group of islands. These two species were recently described as *Alismobates galapagoensis* Pfingstl and Schatz and *Litoribates caelestis* Pfingstl and Schatz, and the latter even represented a new genus. Both species are supposed to be derived from Central or South American coasts. With records from six islands, *A. galapagoensis* shows a wider distribution across the archipelago than *L. caelestis*, which was only collected on two islands (Pfingstl and Schatz 2017). When Schatz (1998) found these mites, he simultaneously investigated the terrestrial oribatid mite fauna of Galápagos and discovered different morphological forms of a single species (e.g. *Scheloribates elegans* Hammer, *Aeroppia adjacens* Mahunka) to be present on different islands or in different habitats of a single island, which indicates ongoing speciation processes. In the intertidal *A. galapagoensis* and *L. caelestis*, on the other hand, signs of diversification or speciation were overseen at first sight as no distinct morphological differences between populations of different islands could be detected and specific morphotypes were absent (Pfingstl and Schatz 2017). However, differentiation at an early stage of speciation may only be expressed in slight size and shape differences, indiscernible for the bare eye. Minto et al. (1991) already demonstrated, with morphometric means, that the widespread Antarctic littoral mite *Halozetes belgicae* (Michael) shows morphological variations between the Antarctic Peninsula and the South Orkney Islands and suggested these geographic variants to be subspecies. A similar but less pronounced situation is given in the assumedly parthenogenetic *Fortuynia hawaiiensis* Pfingstl and Jagersbacher-Baumann from the archipelago of Hawaii. Pfingstl and Jagersbacher-Baumann (2016) analysed different populations morphometrically and could demonstrate slight morphological divergence among four islands.

Given the range of research on interisland variability and speciation in Galápagos (e.g. Finston and Peck 1995; Sequeira et al. 2000; Torres-Carvajal et al. 2014), we hypothesized that *A. galapagoensis* and *L. caelestis* populations show some kind of diversification among the islands of the archipelago though not detected during the first morphological investigation. Therefore, we performed a comprehensive morphometric analysis of several populations from different islands, first, to confirm our hypothesis of ongoing speciation, second, to assess the degree of variation among the islands and third, to infer evolutionary patterns.

## Materials and methods

All analysed *A. galapagoensis* specimens were collected by Heinrich Schatz during four extended expeditions to the Galápagos archipelago between 1982 and 1988 (Schatz 1998) and GAL numbers in parenthesis refer to his collection numbers. In order to get a better estimate of intraspecific variation versus interspecific variation, the assumed closely related *Alismobates inexpectatus* Pfingstl and Schuster was included as outgroup. These specimens were collected on Bermuda by Tobias Pfingstl in 2011 and 2012.

### Investigated populations (Fig. 1)

*Alismobates galapagoensis*—(1) Bartolomé Island, mangroves near Pinnacle Rock (GAL 85-138); littoral zone; leaf litter, sand, mud and moss under *Maytenus octogona*, *Batis maritima* and *Sesuvium edmonstonei*; 12.02.1985; n = 20 (5♀,15♂). (2) Santa Cruz Island, Divine’s Bay (GAL 87-431, GAL 87-432); littoral zone; algae from rocks and mangrove roots (*Rhizophora mangle*); 29.12.1986; n = 4 (3♀, 1♂). (3) San Cristóbal Island, south of wreck Bay (GAL 87-476); littoral zone; decayed leaf litter from *Sesuvium portulacastrum*, *Avicennia germinans* and *Laguncularia racemosa*; 04.01.1987; n = 14 (7♀, 7♂). (4) Isabela island, Punta García, north of vulcan Alcedo (GAL 87-702); arid zone; leaf litter and soil under *Bursera graveolens*, *Cordia lutea* and *C. leucophlyctis*; 21.02.1987; n = 20 (8♀, 12♂).

**Fig. 1:**
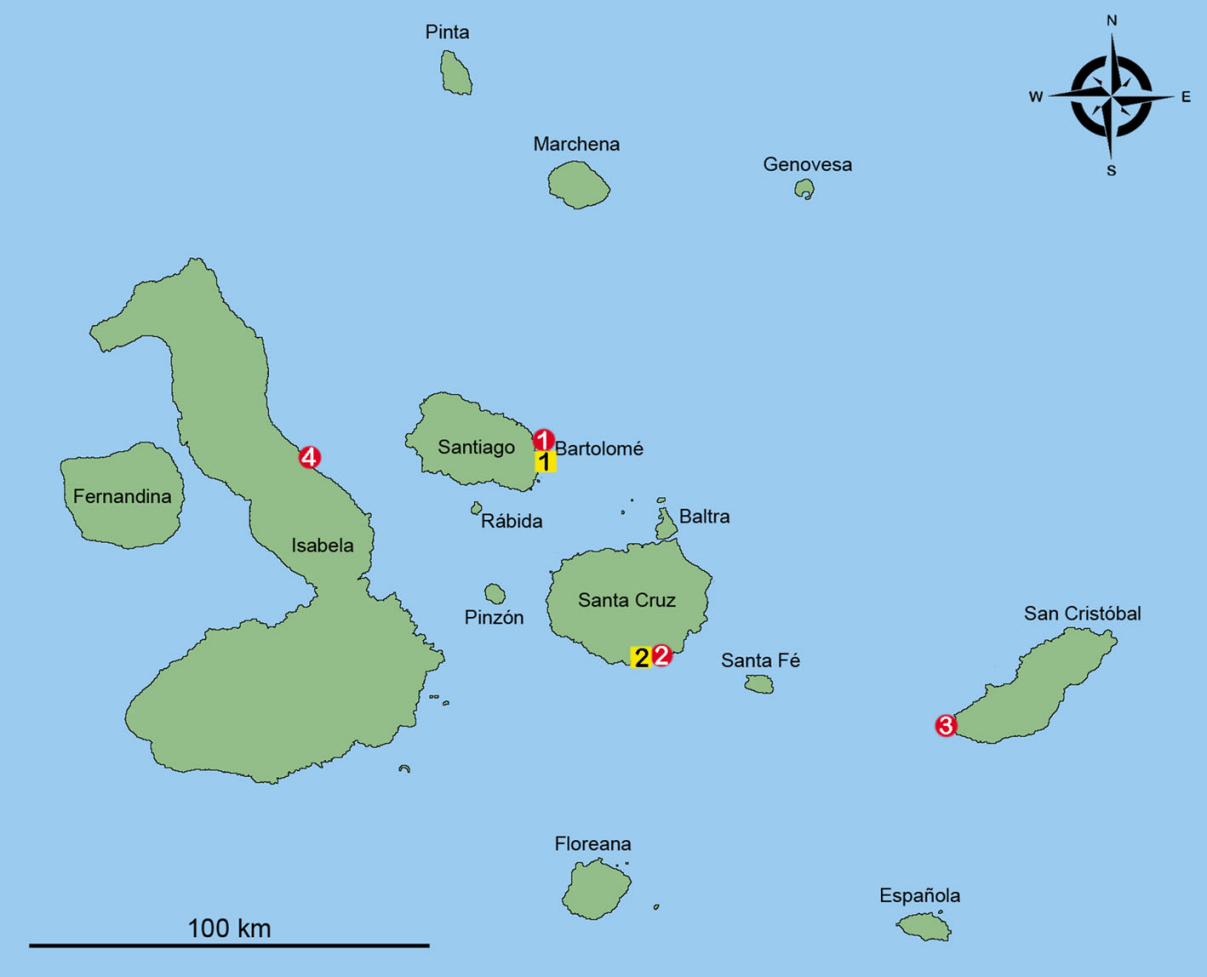
Map of the Galápagos archipelago showing the sample locations of measured *Alismobates galapagoensis* (*circles*) and *Litoribates caelestis* (*square*) populations. *Numbers* refer to location numbers given in text

*Alismobates inexpectatus*—Bermuda, Tobacco Bay; littoral zone; algae (*Bostrychia tenella*) from rocks; 12.04.2012; n = 20 (10♀, 10♂).

*Litoribates caelestis*—(1) Bartolomé Island, mangroves near Pinnacle Rock (GAL 85-137, GAL 87-424); littoral zone; leaf litter, sand, mud and moss under *Maytenus octogona*; 12.02.1985 and 26.12.1986; n = 5 (2♀, 3♂). (2) Santa Cruz Island, Puerto Ayora, near ‘Fragata’ (GAL 87-434); littoral zone; leaf litter and soil under *Rhizophora mangle*; 30.12.1986; n = 15 (7♀, 8♂).

### Measurements and variables

**Fig. 2:**
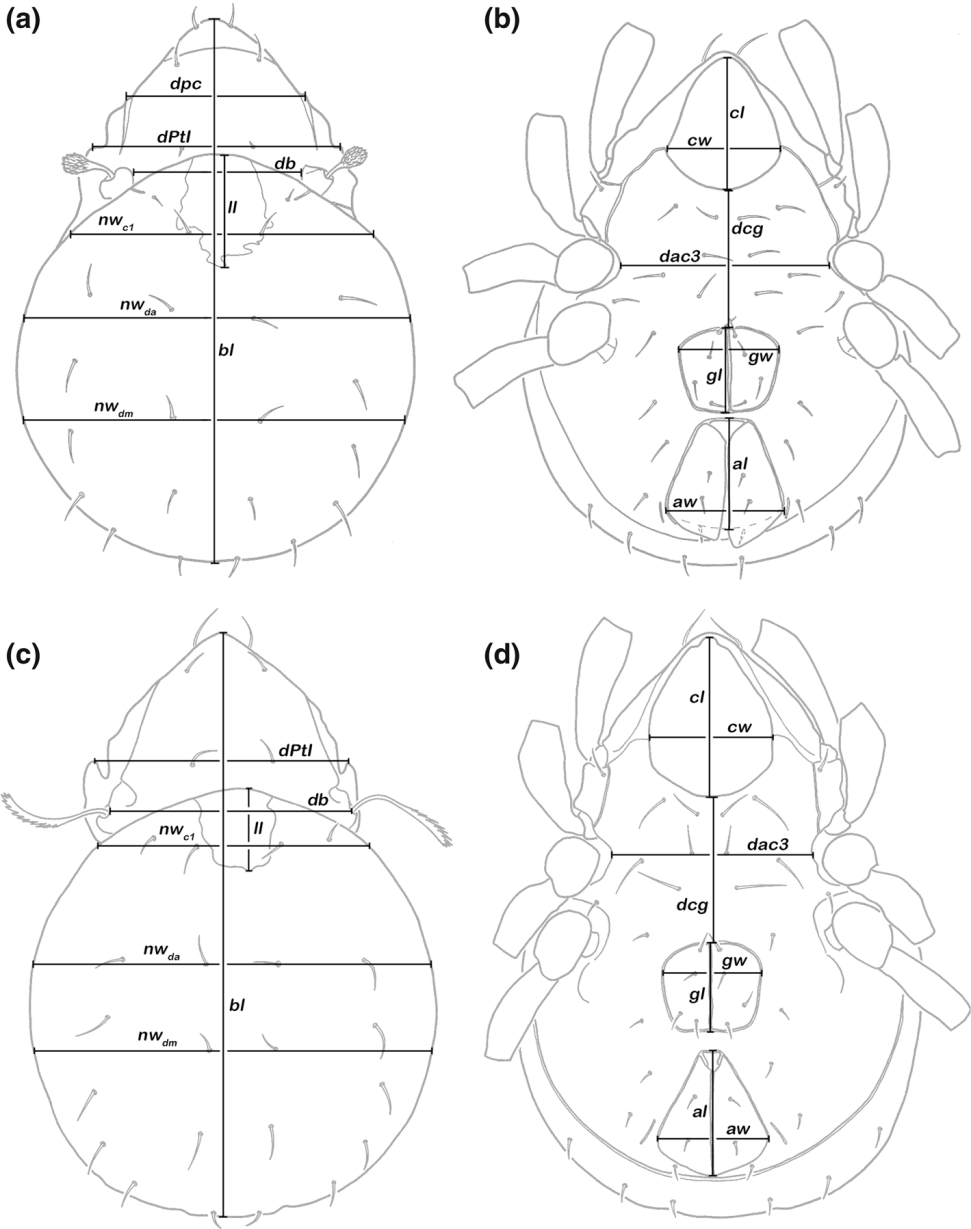
Graphic illustration of measured continuous variables shown on simplified drawings of *Alismobates galapagoensis* (**a**, **b**) and *Litoribates caelestis* (**c**, **d**). Dorsal aspect: *bl*—body length, *dPtI*—distance between pedotecta I, *dpc*—distance between prodorsal carinae, *db*—distance between bothridia, *ll*—lenticulus length, *nw*_*c1*_—notogastral width on level of seta *c*_*1*_, *nw*_*da*_—notogastral width on level of seta *da*, *nw*_*dm*_—notogastral width on level of seta *dm*. Ventral aspect: *cl*—camerostome length, *cw*—camerostome width, *dcg*—distance between camerostome and genital orifice, *dac3*—distance between acetabula 3, *gl*—genital orifice length, *gw*—genital orifice width, *al*—anal opening length, *aw*—anal opening width

Specimens were embedded in lactic acid for temporary slides and measurements were performed using a compound light microscope (Olympus) and ocular micrometre. Sixteen continuous variables were measured in *Alismobates* and 15 in *Litoribates* (Fig. 2).

### Statistical analysis

For univariate statistics, only five randomly chosen specimens out of the 15 available males of *A. galapagoensis* from Bartolomé were used so that each population consisted of an almost equal number of males and females. In all other populations (*A. inexpectatus* from Bermuda, *A. galapagoensis* from Santa Cruz, San Cristóbal and Isabela), all available specimens were used. Mean, minimum, maximum, standard deviation and coefficient of variation (cv) were calculated, and Kruskal–Wallis and Mann–Whitney U test were used for comparing the means of variables between all groups and for pairwise comparisons, respectively.

Multivariate analyses of all specimens listed above were performed on log_10_-transformed raw and size-corrected data. Size correction was done by dividing each variable through the geometric mean of the respective specimen (e.g. Jagersbacher-Baumann 2014; Pfingstl and Jagersbacher-Baumann 2016). In our case, the geometric mean was calculated as the 16th root of the product of all 16 variables measured for each specimen in *Alismobates* and as the 15th root of the product of all 15 variables in *Litoribates*. To reveal patterns of morphological variation, Principal Components Analyses (PCA) were conducted, and to determine the most important variables for differentiating the groups, Canonical Variates Analysis (CVA) was performed in *Alismobates* and Discriminant Analysis (DA) in *Litoribates*.

Preliminary multivariate analyses revealed a size-dependent sexual dimorphism with larger females in all investigated populations. As this sexual dimorphism resulted in overlapping areas in the graphs, males and females of *Alismobates* were analysed separately in PCA and CVA for clarification. The performance of the classification by CVA/DA was tested by calculating the number of specimens correctly classified by all-samples CVA/DA and leave-one-out cross-validation CVA/DA. Multivariate Analysis of Variance (MANOVA) was used for testing the equality of means of all populations of *Alismobates*. To determine phenetic similarities, unrooted neighbour-joining (NJ) trees were constructed using the squared Mahalanobis distances between the populations gained by discriminant analyses based on raw and size-corrected data (Kerschbaumer et al. 2011; Jagersbacher-Baumann 2015). For testing equality of means and for constructing the NJ-trees, males and females were again pooled together. All analyses were performed with PAST 3.11 (Hammer et al. 2001).

## Results

### *Alismobates* populations

#### Descriptive/univariate statistics

Univariate analyses of *A. inexpectatus* from Bermuda and *A. galapagoensis* from Bartolomé, Santa Cruz, San Cristóbal and Isabela revealed significant differences in all variables between all five populations (Kruskal–Wallis test: *p* < 0.01) (Table 1). Pairwise comparisons of populations by Mann–Whitney U test showed that most differences existed between *A. galapagoensis* populations from San Cristóbal versus Bartolomé and from San Cristóbal versus Isabela, and between *A. inexpectatus* (from Bermuda) versus *A. galapagoensis* from Bartolomé as well as between *A. inexpectatus* (Bermuda) versus *A. galapagoensis* from Isabela. Among all analysed populations, *A. inexpectatus* was by trend largest as it had the highest values for most of the measured variables. When *A. inexpectatus* was removed from the analysis, Kruskal–Wallis still revealed significant differences (*p* < 0.05) between the remaining populations from Galápagos in all variables except *dac3*. Within the *A. galapagoensis* populations, the specimens from San Cristóbal were largest and those from Bartolomé were smallest. The variability of characters as indicated by cv was moderate with a maximum cv value of 0.10 in all populations. In all populations, highest cv values (between 0.07 and 0.10) were calculated for either *gl*, *gw* or both, and both these variables are directly related to gender.

**Table 1:** Mean (x), minimum–maximum (min–max) in µm, standard deviation (sd) and coefficient of variation (cv) of *Alismobates inexpectatus* from Bermuda and four *A. galapagoensis* populations from Galápagos

	Bermuda (n = 20)			Bartolomé (n = 10)			Santa Cruz (n = 4)		
	x (min–max)	sd	cv	x (min–max)	sd	cv	x (min–max)	sd	cv
*bl*	383.85 (351–425)	21.26	0.06	355.40 (326–397)	23.10	0.06	375.75 (351–404)	24.88	0.07
*dpc*	112.75 (105–120)	3.77	0.03	102.00 (99–105)	2.83	0.03	108.75 (105–114)	3.77	0.03
*dPtI*	164.60 (157–170)	3.59	0.02	151.15 (148–154)	2.45	0.02	157.75 (154–163)	3.77	0.02
*db*	101.40 (92–108)	4.20	0.04	98.00 (95–102)	2.83	0.03	105.50 (102–111)	4.36	0.04
*ll*	76.25 (65–86)	5.66	0.07	68.30 (65–71)	2.63	0.04	77.00 (74–83)	4.24	0.06
*nw*_*c1*_	199.25 (182–231)	13.31	0.07	192.50 (182–203)	7.11	0.04	210.75 (200–219)	7.89	0.04
*nw*_*da*_	250.25 (234–225)	10.04	0.04	234.30 (225–246)	7.13	0.03	258.00 (243–265)	10.39	0.04
*nw*_*dm*_	244.45 (225–262)	10.30	0.04	230.90 (215–246)	10.17	0.04	251.00 (237–262)	11.63	0.05
*cl*	103.30 (86–111)	5.62	0.05	102.30 (99–108)	2.98	0.03	104.25 (102–105)	1.50	0.01
*cw*	77.60 (74–80)	1.85	0.02	75.20 (71–80)	2.53	0.03	75.50 (74–77)	1.73	0.02
*dcg*	85.10 (77–95)	4.47	0.05	71.60 (68–77)	3.10	0.04	79.25 (74–83)	3.77	0.05
*dac3*	136.90 (129–148)	4.47	0.03	122.40 (120–126)	2.37	0.02	126.00 (120–132)	5.48	0.04
*gl*	61.20 (55–68)	5.20	0.08	56.90 (52–65)	4.77	0.08	65.50 (59–71)	4.93	0.08
*gw*	79.50 (71–86)	4.93	0.06	69.70 (65–77)	5.36	0.08	77.25 (71–80)	4.27	0.06
*al*	91.15 (83–95)	3.27	0.04	85.10 (83–92)	3.18	0.04	89.00 (89–89)	0.00	0.00
*aw*	79.70 (74–86)	2.74	0.03	79.10 (65–74)	2.47	0.04	75.50 (71–80)	3.87	0.05

	San Cristóbal (n = 14)			Isabela (n = 20)			KW	KW Galápagos	MWU
	x (min–max)	sd	cv	x (min–max)	sd	cv			
*bl*	382.00 (348–425)	22.73	0.06	358.80 (326–396)	19.36	0.05	***	***	*** a, b, d, ** c
*dpc*	111.00 (102–123)	5.39	0.05	103.80 (95–111)	4.38	0.04	***	***	*** a, b, ** c, d
*dPtI*	164.50 (160–169)	3.28	0.02	155.05 (148–163)	3.80	0.02	***	***	*** a, b, c, d
*db*	107.93 (102–114)	3.91	0.04	103.10 (99–111)	3.63	0.04	***	***	*** c, ** g, * d, f
*ll*	82.14 (71–89)	5.70	0.07	73.25 (68–80)	3.63	0.05	***	***	*** c, d, ** a, * e, f
*nw*_*c1*_	206.36 (188–231)	11.07	0.05	188.80 (175–203)	7.42	0.04	***	***	*** d, * c, h
*nw*_*da*_	256.29 (237–271)	9.56	0.04	234.15 (219–249)	8.29	0.04	***	***	*** b, c, d, ** a, * h
*nw*_*dm*_	251.86 (231–271)	11.07	0.04	231.30 (219–249)	8.59	0.04	***	***	*** d, ** b, c
*cl*	106.64 (102–111)	2.24	0.02	103.00 (99–105)	2.25	0.02	**	***	** d, * c
*cw*	77.57 (74–82)	1.91	0.02	73.75 (71–80)	2.69	0.04	***	**	*** b,** d
*dcg*	77.07 (71–86)	3.85	0.05	71.00 (65–77)	3.23	0.05	***	***	*** a, b, g, **d, * h
*dac3*	122.36 (117–126)	2.92	0.02	122.20 (117–129)	2.89	0.02	***		*** a, b, g, * i
*gl*	65.71 (60–71)	3.63	0.06	59.85 (52–68)	5.83	0.10	**	**	** c
*gw*	75.43 (68–83)	5.50	0.07	71.50 (62–80)	6.65	0.09	***	*	** a, b
*al*	90.50 (86–95)	2.56	0.03	86.10 (80–92)	3.31	0.04	***	***	*** b, ** a, d, * c
*aw*	78.07 (74–92)	4.80	0.06	73.05 (68–80)	2.91	0.04	***	***	*** a, b, c, ** d

Results of Kruskal–Wallis (KW) and Mann–Whitney-U (MWU) tests are given, * 0.01 < *p* < 0.05, ** 0.001 < *p* < 0.01, *** *p* < 0.001. a = Bermuda versus Bartolomé, b = Bermuda versus Isabela, c = Bartolomé versus San Cristóbal, d = San Cristóbal versus Isabela, e = Bartolomé versus Santa Cruz, f = Bartolomé versus Isabela, g = Bermuda versus San Cristóbal, h = Santa Cruz versus Isabela and i = Bermuda versus Santa Cruz

#### Multivariate analyses

**Fig. 3:**
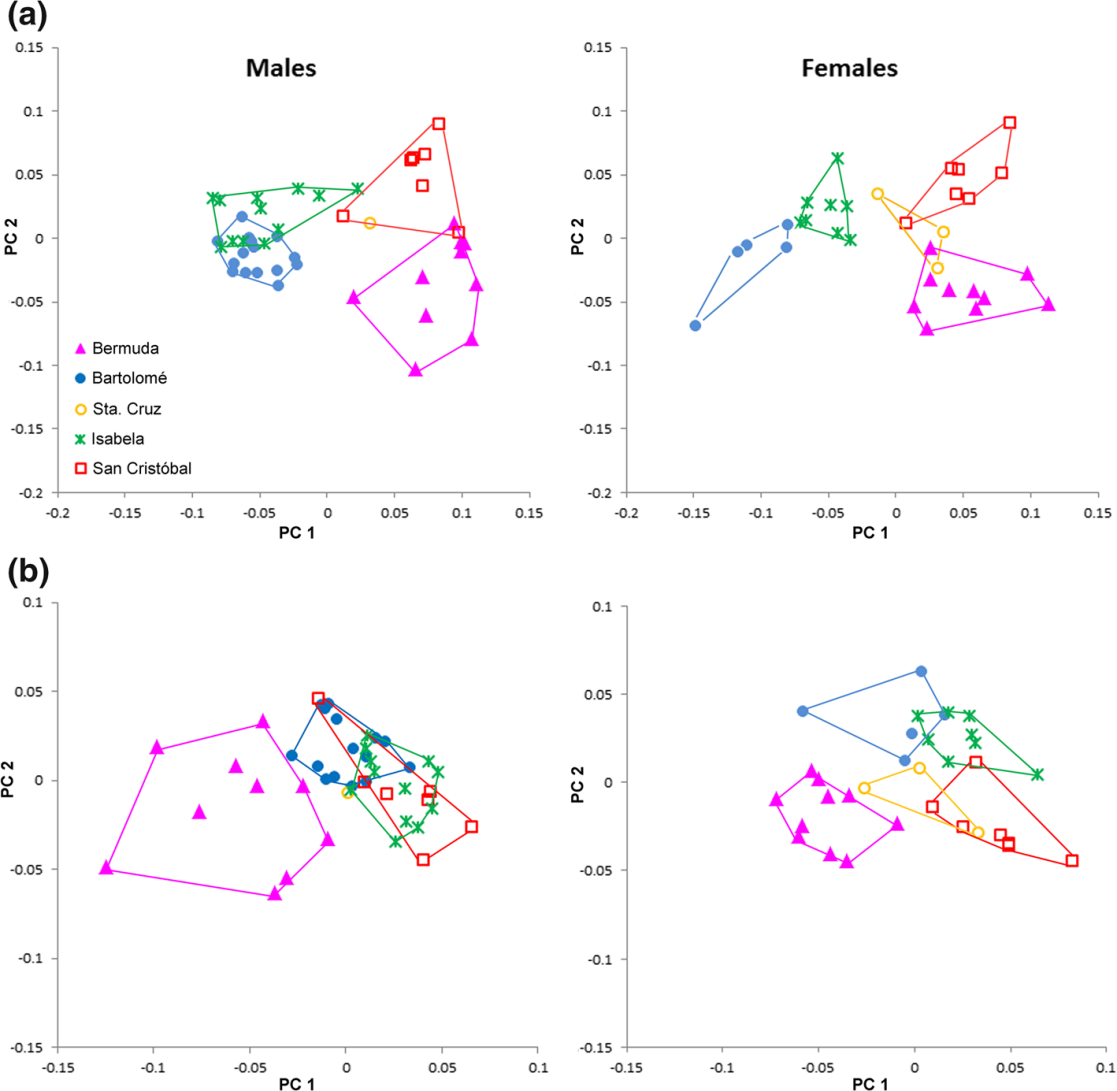
Scatter plots gained from PCA on log_10_-transformed **a** raw data and **b** size-corrected data of *Alismobates inexpectatus* from Bermuda (a different species) and four *A. galapagoensis* populations from Galápagos

PCA on log-transformed raw data resulted in a separation of the analysed populations in females as well as in males, but in males there were more overlapping areas than in females (Fig. 3a). The best separation was given by a combination of PC1 and PC2, which together explained 72.69% of the total variation in males (PC1 55.60%, PC2 17.09%) and 70.50% in females (PC1 51.71%, PC2 18.79%). In both cases, all variables on PC1 had positive loadings, a fact that already hints to a correlation between PC1 and size. A strong correlation was proven when the PC1 values of each specimen were plotted against the respective geometric means (r = 0.99 in both males and females). In accordance with the results from the univariate statistic, *A. inexpectatus* from Bermuda and *A. galapagoensis* populations from San Cristóbal and Santa Cruz were populations with larger individuals whereas *A. galapagoensis* populations from Isabela and Bartolomé consisted of smaller specimens. Females from Isabela and Bartolomé were separated from each other on PC1 whereas males of the respective populations overlapped. In males as well as in females, PC2 at least partly separated the *A. galapagoensis* populations from Isabela and San Cristóbal from the population from Bartolomé and from *A. inexpectatus*. The variables with highest loadings were *dcg* for males and *ll* for females on PC1 and *ll* and *dcg* for males and *dcg*, *dac3* and *ll* for females on PC2.

After size correction, the *A. galapagoensis* males of all populations from Galápagos clustered together, but there was still a separation, albeit with overlaps, between the females (Fig. 3b). The decrease of total variation after size correction was 49.93% in males and 44.69% in females. Males as well as females of *A. inexpectatus* were separated from the *A. galapagoensis* populations by a combination of PC1 and PC2, and the males of *A. inexpectatus* showed a remarkable variability on both axes. In males, PC1 and PC2 together explained 54.49% of the total variation (PC1 37.74%, PC2 16.76%), and variables with highest loadings were *dcg* on PC1 and *ll* on PC2. In females, PC1 and PC2 together explained 51.96% of the total variation (PC1 34.18%, PC2 17.79%). In *A. galapagoensis* populations, PC1 separated the population from Bartolomé and Santa Cruz from those from San Cristóbal and Isabela, and PC2 separated the populations from San Cristóbal and Santa Cruz from those from Isabela and Bartolomé. The variables *dcg* and *dac3* were the ones with the highest loadings on PC1, and *ll* on PC2.

**Fig. 4:**
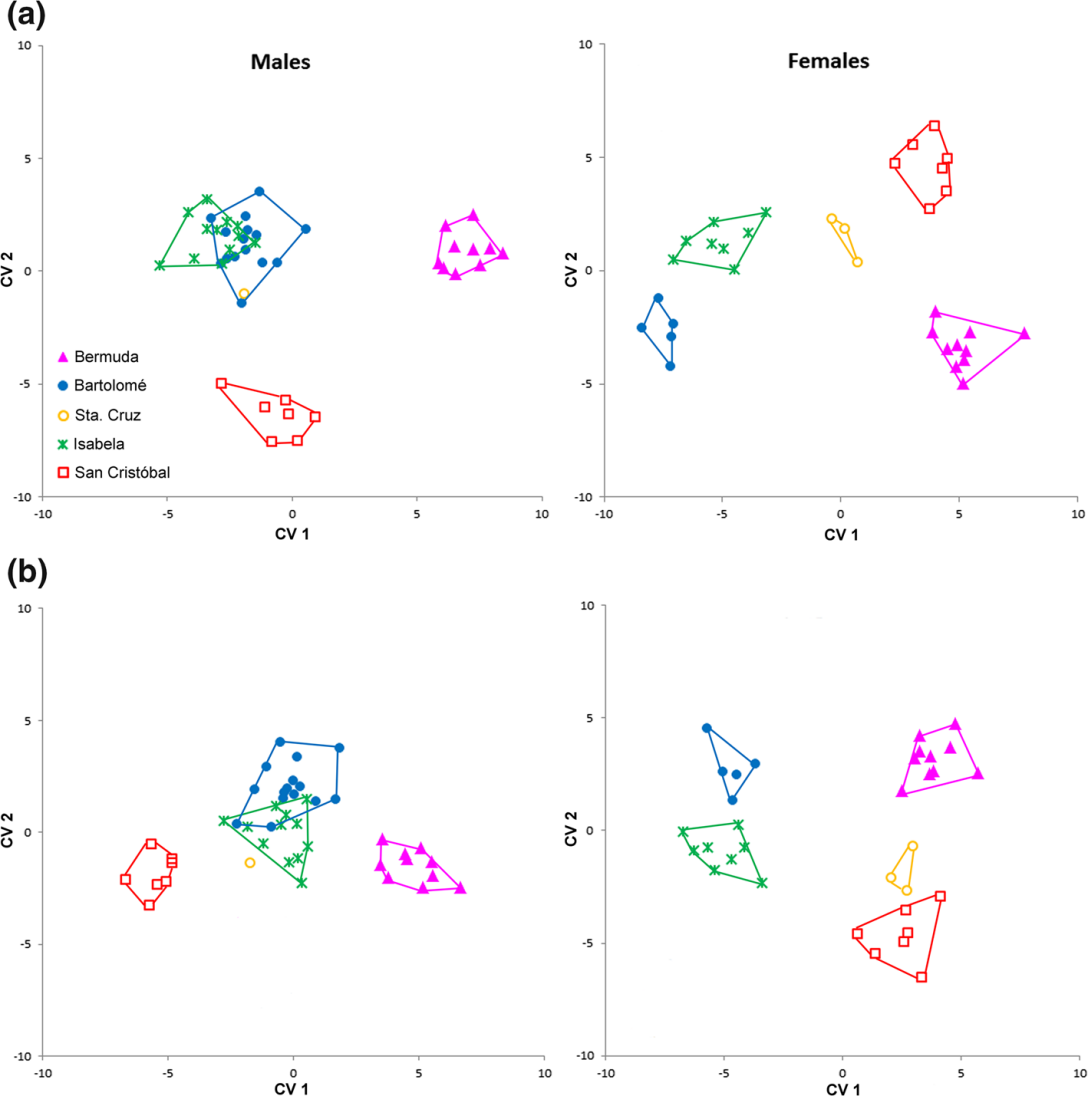
Scatter plots gained from CVA on log_10_-transformed **a** raw data and **b** size-corrected data of *Alismobates inexpectatus* from Bermuda (a different species) and four *A. galapagoensis* populations from Galapagos

In males, CVA on both, log-transformed raw and size-corrected data, revealed a partial separation of the populations: *Alismobates inexpectatus* and *A. galapagoensis* from San Cristóbal were always well separated from each other and from the remaining *A. galapagoensis* populations, which clustered together (Fig. 4). In CVA on raw data, CV1 explained 59.16% of the total variation and CV2 explained 31.64%. Classification by CVA revealed that 97.78% of all specimens could be correctly classified in all-samples CVA and 80.00% in leave-one-out cross-validated (LOO-CV) CVA. Misclassified specimens almost exclusively belonged to the *A. galapagoensis* populations from Santa Cruz, Isabela and Bartolomé. *Alismobates inexpectatus* was separated from the other populations on CV1, and the variables contributing most to variation on this axis were *dcg*, *dac3* and *gw*. CV2 separated *A. galapagoensis* from San Cristóbal from the other populations, and variables with high loadings on this axis were *ll* and *gl* (Table 2). After size correction, CV1 explained 69.73% and CV2 17.65% of the total variation, and the power of classification by CVA slightly weakened: the percentage of correctly classified specimens was 97.78% in all-samples CVA and still 75.56% in LOO-CV CVA. Again, misclassified specimens mostly belonged to the populations from Santa Cruz and Bartolomé. CV1 separated *A. inexpectatus* and *A. galapagoensis* from San Cristóbal from each other as well as from the remaining populations, and the variable with the highest loading on this axis was *dac3*. On CV2, *A. galapagoensis* from Bartolomé was separated from the other populations, with an overlap with the population from Isabela. Variables contributing most to variation on CV2 were *ll*, *cl* and *cw*. MANOVA revealed highly significant (*p* < 0.001) differences between all populations in raw data as well as in size-corrected data.

**Table 2:** Loadings of the two canonical axes CV1 and CV2 for CVA on one population of *Alismobates inexpectatus* from Bermuda and four populations of *A. galapagoensis* from Galápagos

	Males				Females			
	Raw data		Size-corrected data		Raw data		Size-corrected data	
	CV1	CV2	CV1	CV2	CV1	CV2	CV1	CV2
*bl*	0.003	−0.003	0.000	0.000	0.002	0.000	−0.001	0.000
*dpc*	0.003	−0.002	0.001	0.001	0.004	0.000	0.001	0.000
*dPtI*	0.003	−0.003	0.000	−0.001	0.003	0.000	0.000	0.000
*db*	0.000	−0.004	−0.003	0.002	0.001	0.004	−0.003	−0.003
*ll*	0.002	−**0.006**	−0.003	−**0.005**	**0.005**	**0.005**	0.002	−**0.005**
*nw*_*c1*_	0.001	−0.003	−0.002	0.004	0.003	0.002	0.001	−0.001
*nw*_*da*_	0.002	−0.004	−0.001	0.000	0.003	0.002	0.001	−0.001
*nw*_*dm*_	0.003	−0.004	0.000	−0.001	0.002	0.003	−0.001	−0.002
*cl*	0.000	−0.002	−0.002	**0.005**	0.000	0.002	−0.003	−0.001
*cw*	0.002	−0.002	0.000	**0.005**	0.001	0.000	−0.002	0.001
*dcg*	**0.008**	−0.002	0.004	−0.003	**0.005**	−**0.005**	**0.004**	**0.005**
*dac3*	**0.005**	0.001	**0.005**	0.002	0.003	−**0.005**	0.000	**0.006**
*gl*	0.001	−**0.007**	−0.004	−0.002	0.002	0.004	−0.001	−0.003
*gw*	**0.006**	−0.002	0.003	−0.003	0.003	−0.001	0.000	0.001
*al*	0.003	−0.003	0.000	0.001	0.002	0.000	−0.001	0.001
*aw*	0.004	−0.003	0.001	−0.004	0.004	0.001	0.002	0.000

High loadings explaining differences between the groups are given in bold

CVA on both log-transformed raw and size-corrected data of females resulted in a separation of all populations (Fig. 4). In CVA on raw data, CV1 explained 64.37% and CV2 explained 23.76% of the total variation. On CV1, the *A. galapagoensis* populations from Isabela and Bartolomé were separated from the population from Santa Cruz and also from the population from San Cristóbal and *A. inexpectatus*. CV2 showed a separation between *A. galapagoensis* from Isabela, Santa Cruz and San Cristóbal from *A. galapagoensis* from Bartolomé and *A. inexpectatus*. Variables contributing most to variation on the respective axes were *ll* and *dcg* on CV1 and *ll*, *dcg* and *dac3* on CV2 (Table 2). In all-samples CVA, 100% of all specimens could be classified correctly, and in LOO-CV CVA this number dropped to 87.88%. After size correction, CV1 explained 56.75% and CV2 33.62% of the total variation. CV1 separated *A. galapagoensis* from Isabela and Bartolomé from the remaining populations, and CV2 separated *A. inexpectatus* from all *A. galapagoensis* populations, except for Bartolomé. Variables with high loadings were *dcg* on CV1 and *ll*, *dcg* and *dac3* on CV2 (Table 2). The percentage of correctly classified specimens dropped to 90.91% in all-samples CVA and 78.79% in LOO-CV CVA. Misclassified specimens were found mostly in *A. inexpectatus* and *A. galapagoensis* from Isabela. MANOVA revealed highly significant (*p* < 0.001) differences between all populations in raw data as well as in size-corrected data.

**Fig. 5:**
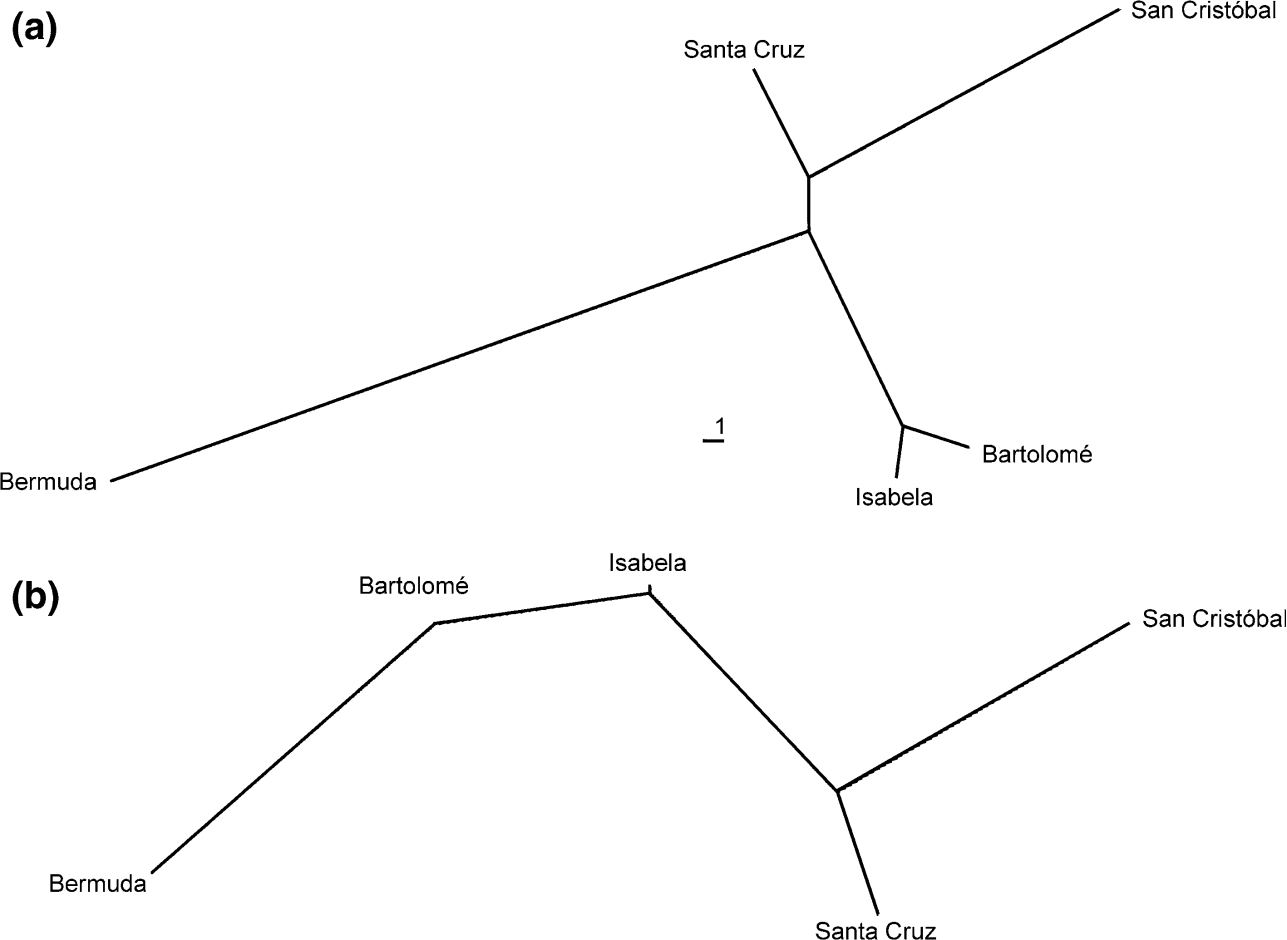
Unrooted neighbour-joining (NJ) trees of *Alismobates inexpectatus* from Bermuda and four *A. galapagoensis* populations from Galapagos based on squared Mahalanobis distances obtained from canonical variates analysis (CVA) on canonical variates values of log_10_-transformed **a** raw data and **b** size-corrected data

The unrooted NJ trees based on phenetic similarities revealed that raw data showed a better separation between populations than size-corrected data (Fig. 5). In the tree based on raw data, *A. inexpectatus* was clearly separated from all *A. galapagoensis* populations. Within *A. galapagoensis*, the populations from Isabela and Bartolomé were closest together, and the population from San Cristóbal was most different from the remaining populations (Fig. 5a). A very similar pattern was still recognizable in size-corrected data, but the distances between each population are larger (Fig. 5b).

### *Litoribates* populations

#### Descriptive/univariate statistics

There are few significant differences between the two populations from Bartolomé and Santa Cruz (Table 3), only the two variables describing the camerostome *cl* and *cw* differ significantly (*p* < 0.05).

**Table 3:** Mean (x), minimum–maximum (min–max) in µm, standard deviation (sd) and coefficient of variation (cv) of two *Litoribates caelestis* populations from Galápagos

	Bartolomé (n = 5)			Santa Cruz (n = 15)			MWU
	x (min–max)	sd	cv	x (min–max)	sd	cv	
*bl*	324.20 (307–342)	13.31	0.04	320.93 (308–336)	8.97	0.03	
*dPtI*	145.00 (142–148)	3.00	0.02	142.60 (139–148)	2.59	0.02	
*db*	129.60 (126–135)	3.29	0.03	128.80 (126–132)	2.40	0.02	
*ll*	48.40 (46–52)	3.29	0.07	50.60 (43–55)	2.97	0.06	
*nw*_*c1*_	150.40 (145–160)	6.50	0.04	154.33 (132–172)	9.77	0.06	
*nw*_*da*_	200.60 (191–212)	7.77	0.04	204.20 (194–214)	6.41	0.03	
*nw*_*dm*_	199.40 (188–212)	9.10	0.05	201.80 (191–215)	5.98	0.03	
*cl*	90.80 (80–95)	6.22	0.07	96.00 (89–99)	3.00	0.03	*
*cw*	69.80 (65–71)	2.68	0.04	67.27 (65–69)	1.44	0.02	*
*dcg*	80.60 (71–92)	7.77	0.10	73.53 (66–80)	3.60	0.05	
*dac3*	108.60 (105–114)	3.91	0.04	110.07 (108–112)	1.53	0.01	
*gl*	47.20 (43–49)	2.68	0.06	44.93 (40–49)	3.06	0.07	
*gw*	54.00 (49–59)	3.74	0.07	54.07 (49–62)	4.56	0.08	
*al*	70.00 (66–74)	3.08	0.04	69.00 (65–71)	1.85	0.03	
*aw*	53.80 (52–55)	1.64	0.03	55.13 (52–59)	2.29	0.04	

Results of Mann–Whitney-U (MWU) test are given, * *p* < 0.05

#### Multivariate analyses

**Fig. 6:**
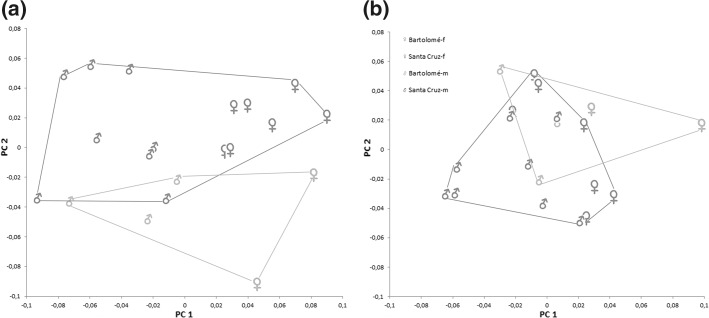
Scatter plots gained from PCA on log_10_-transformed **a** raw data and **b** size-corrected data of *Litoribates caelestis* populations from Bartolomé and Santa Cruz

PCA on log-transformed raw data resulted in a partial separation of the two populations (Fig. 6a). The combination of PC1 and PC2 explained 69.82% of the total variation (PC1 47.30%, PC2 22.52%). PC1 was strongly correlated with size defined as the geometric mean (r = 0.99) and there was a clear variation present on this component. Thus, all specimens with high PC1 values were large individuals, and all of them were females. There was a clear separation between males and females along PC1, indicating a sexual dimorphism connected with overall size. The two populations were separated mainly from each other on PC2. Variables contributing most to variation on this component and thus to the separation between the populations were *ll* and *dcg*. The separation between males and females on PC1 was clearer than the separation between the two populations on PC2. After size correction, which resulted in a reduction of 32.88% of the total variation, the two populations as well as the two sexes overlapped in PCA (Fig. 6b).

Discriminant analyses (DA) divided the two populations into two groups in both raw and size-corrected data. Variables with highest loadings, thus mainly responsible for separation of the two groups, were *dcg* in raw data and *dcg*, *cl* and *gw* in size-corrected data. However, the groups did not significantly differ from each other as Bonferroni corrected Hotelling’s T^2^ tests revealed no significant differences (*p* = 0.15 in raw data and *p* = 0.67 in size-corrected data). Accordingly, a jack-knifed discriminant analysis was only able to classify 65 and 60% of the specimens correctly in raw data and size-corrected data, respectively.

## Discussion

### General aspects

Describing *A. galapagoensis*, Pfingstl and Schatz (2017) already noticed a conspicuous morphological similarity to the Bermudian *A. inexpectatus*, suggested a close relationship of the two species and further stated that *A. galapagoensis* may be derived from Central or South American shores. In the meantime, *Alismobates* specimens strongly resembling *A. inexpectatus* were found on the Caribbean coast of Panama (Pfingstl, unpublished data). Whether these specimens belong to *A. inexpectatus* or do represent another sister species of *A. galapagoensis* cannot be answered yet and will be part of future investigations. However, this record clearly fills the large geographic gap between Galápagos and Bermuda and supports the idea that *A. galapagoensis* may have a Central American origin (Pfingstl and Schatz 2017). Several studies propose that colonization of Galápagos by different organisms happened via passive drift on the Humboldt Current, which transports cool waters from the South American coast to the archipelago (e.g. Sequeira et al. 2000; Caccone et al. 2002; Torres-Carvajal et al. 2014). Considering their presently known distribution, we presume that the investigated mites probably reached Galápagos via the Panamá Current, which carries warm tropical waters from Central America (Lea et al. 2006) and fuses with the Peru Coastal Current before arriving at Galápagos.

Despite the similarity between *A. inexpectatus* and *A. galapagoensis*, the present morphometric analysis clearly separates both species and hence confirms their distinctness. The present study also revealed the presence of a slight sexual dimorphism concerning body size in all investigated species. Females are by trend larger and possess a relatively larger genital opening than males. This is not unusual as a small degree of sexual dimorphism is common among most oribatid mites with females being slightly larger and showing larger genital plates than males; only 1% of all species show explicit differences between the genders (e.g. Behan-Pelletier 2015). However, a rather unusual result of the present study is that females of *A. galapagoensis* from different islands show higher variation in shape than the respective males. As can be seen in Fig. 3b, females from different populations are clearly separated from each other, whereas the according males cluster. The reason for this sex related difference in shape variation is presently unknown and remains puzzling.

### Variation among islands

The two *Litoribates* populations from Bartolomé and Santa Cruz did not show significant differences in their variables neither in univariate nor in multivariate analysis, although a slight separating trend can be observed in the PCA graphs. Bartolomé and Santa Cruz lie only approx. 30 km apart and both populations were found in similar mangrove leaf litter habitats, accordingly the present result is not surprising.

The *Alismobates* populations from four different islands of the Galápagos archipelago, on the other hand, showed significant differences in all univariate and multivariate analyses. Although size-correction decreased the total variation, the populations could still be separated from each other (at least in females), which strongly points to genetically induced variation (e.g. Stekolnikov and Klimov 2010). In the raw data, size differences with individuals from San Cristóbal being the largest and specimens from Bartolomé and Isabela being the smallest were evident. Pfingstl and Jagersbacher-Baumann (2016) demonstrated similar variations among Hawaiian *F. hawaiiensis* populations, whereas they could not explain if these divergences represented cases of phenotypic plasticity or cases of diverging genomes. In the present case an assessment is also difficult to make because our knowledge about ecological factors in the microhabitat and their influence on phenotypic plasticity in these mites is limited. Nevertheless, the size variation of the Galápagos *Alismobates* populations shows a clear gradient from East (large) to West (small) which rather points to directional diverging genomes than to non-genetic intraspecific variation.

Apart from the overall size differences, the *Alismobates* populations also show several significant differences in single variables. The mainly affected variables are the lenticulus length, the distance between camerostome and genital orifice and the distance between acetabula III. The latter two variables are properties of the epimeral area which indicates that this whole region is subject to strong variation. Why these traits vary between the island populations is unknown and could only be speculated at this point in time.

**Fig. 7:**
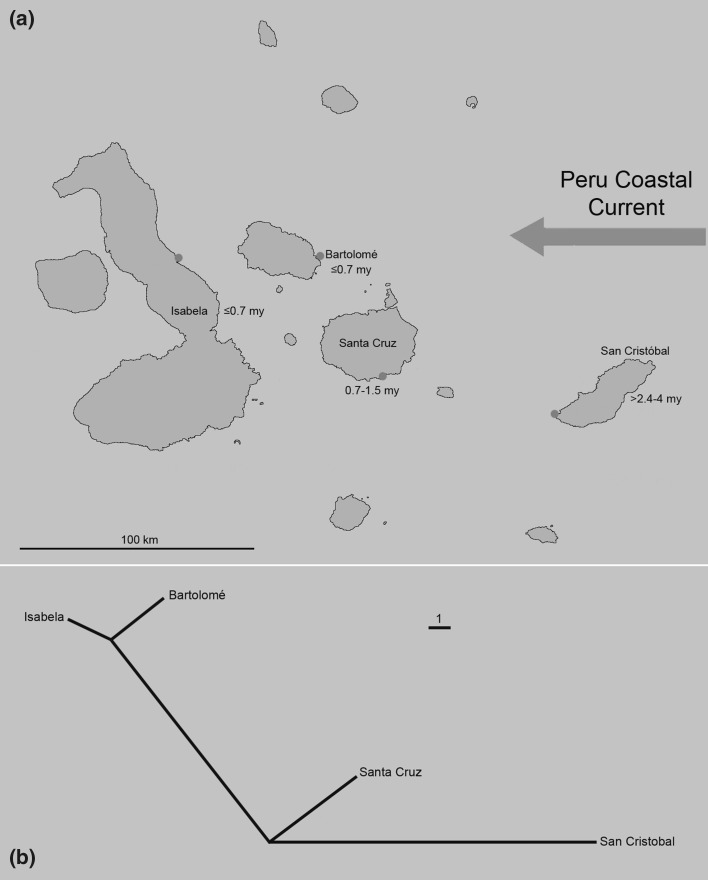
Biogeographical aspects of *Alismobates galapagoensis* populations. **a** Map of the Galápagos archipelago showing approximate geological ages of the islands (after Simkin 1984) and location of investigated populations. **b** Spatially modified unrooted NJ-tree based on squared Mahalanobis distances obtained from canonical variates analysis (CVA) (raw data) reflecting similarities between geography and morphological differentiation

Another interesting result of the present morphometric study is that the morphological divergence between the various *Alismobates* populations seems to correlate with the geographic distance between the islands (compare Fig. 7). The larger the distance between the islands, the larger are the morphological differences between the respective populations. Considering gene flow between islands, this would mean genetic exchange has happened less frequently between populations of distant islands than between closer located islands. This result together with the clear gradient in size indicates that colonization of the islands might have happened only in one direction, from East to West. A similar colonization route has also been proposed for other flightless animals, for example weevils (Sequeira et al. 2000), geckos (Torres-Carvajal et al. 2014) or giant tortoises (Caccone et al. 2002).

This leads us to the question of how littoral mites can move from one landmass to the other. Schatz (1991) already discussed possible agents for dispersal of mites to remote islands in detail and stated that there are mainly three modes: first, rafting on ocean currents, second, transport by other organisms (mainly birds) and third, introduction by humans. However, birds and ships can travel fast, arbitrarily and in all directions between the islands of the Galápagos archipelago and if mites would mainly be transported by these, gene flow would not be restricted considerably by distance and consequently morphological variance would be more balanced across the archipelago. Ocean currents, on the other hand, are not directly linear and relatively slow moving dispersal agents therefore the chance of surviving this mode of transport clearly decreases with distance. According to this, gene flow between far distant populations happens more seldom and subsequently leads to stronger diverging genomes and morphologies. One still might argue that the same may apply to transport by birds or by wind, but the investigated mites do not show any behavioural or morphological adaptations to phoresy (e.g. Pfingstl 2017) and wind dispersal has mainly been shown in mites associated with tree habitats (e.g. Lehmitz et al. 2011). Pfingstl (2017) already reviewed data on dispersal mechanisms of intertidal oribatid mites and came to the conclusion that hydrochory, i.e. transport along ocean currents, is the most likely mode of long distance transport at least for fortuyniid mites. The observed correlation between geographic distance and morphological divergence may be another indication that these intertidal mites have been predominantly dispersed throughout Galápagos by drifting on ocean currents. Recent prevailing surface currents within the archipelago basically run in a north-westerly direction (Caccone et al. 2002), which would be congruent with the above mentioned theory. Without molecular genetic data it is not yet possible to assess the degree of gene flow between the island populations and to tell how far this diversification has actually proceeded on a genetic base. However, present data clearly show morphological divergence among the different populations which we believe is a result of an ongoing speciation process. Furthermore, we think that the correlation between morphological divergence and geographic distance is mainly a result of hydrochorous dispersal between the islands.
